# Evaluation of Two Dry Commercial Therapeutic Diets for the Management of Feline Chronic Gastroenteropathy

**DOI:** 10.3389/fvets.2017.00069

**Published:** 2017-05-10

**Authors:** Sally C. Perea, Stanley L. Marks, Leighann Daristotle, Patricia E. Koochaki, Richard Haydock

**Affiliations:** ^1^P&G Pet Care, Mason, OH, USA; ^2^Department of Medicine and Epidemiology, School of Veterinary Medicine, University of California at Davis, Davis, CA, USA; ^3^Waltham Centre for Pet Nutrition, Melton Mowbray, Leicestershire, UK

**Keywords:** cat, gastropathy, enteropathy, gastrointestinal, vomiting, diet, nutrition

## Abstract

Management of feline chronic gastroenteropathies has included intervention with both veterinary therapeutic formulas designed to manage non-specific gastrointestinal disorders and those designed with limited novel or hydrolyzed ingredients for management of food-responsive enteropathies and steroid-responsive enteropathies (inflammatory bowel disease). There have been few studies evaluating the use of dietary intervention for the management of feline chronic gastroenteropathy. This prospective, multi-center study evaluated the use of two commercially available feline veterinary therapeutic dry diets designed to manage non-specific gastrointestinal disorders in 28 cats with a history of chronic vomiting and/or diarrhea. The majority of cats enrolled in the study had a history of vomiting (*n* = 25), with a smaller number having a history of concurrent diarrhea (*n* = 2) or diarrhea alone (*n* = 3). Cats were excluded if diagnostic tests identified any systemic or infectious disease that could be associated with the clinical signs of vomiting or diarrhea, and if they were panhypoproteinemic, hypoalbuminemic, hypocobalaminemic, or had a Spec fPL ≥5.4 µg/L. Cats were randomized to one of two veterinary therapeutic diets for 4 weeks. Feeding of both therapeutic diets resulted in a numeric reduction in the number of vomiting episodes over the 4-week period, but no significant differences were seen between dietary interventions. When looking within dietary groups, significant differences were seen in cats fed Diet A with reductions of 69.1, 73.3, and 63.2% (*p* values of 0.008, 0.003, and 0.029) in weeks 2, 3, and 4, respectively, when compared to week 0. The probability of vomiting also showed significant reductions in cats fed Diet A between weeks 0 and 2, 3, and 4, with odds ratios of 0.008, 0.005, and 0.005, respectively (*p* values of 0.038, 0.23, and 0.23). Results of this study demonstrate that a veterinary therapeutic gastrointestinal formula can be effective in the management of feline chronic vomiting. Cats that fail to respond to this dietary approach after a 2- to 4-week trial may benefit from a limited novel or hydrolyzed ingredient formula and may require additional diagnostics to better characterize the underlying disease.

## Introduction

Dietary recommendation for the management of chronic gastroenteropathy in cats has included the use of both veterinary therapeutic elimination and veterinary therapeutic gastrointestinal formulas (1–3). Veterinary elimination diets are designed to limit dietary antigens by incorporating uncommon or hydrolyzed protein ingredients and are utilized to diagnose and manage gastrointestinal and/or dermatologic conditions with an underlying food intolerance or food allergy. Veterinary gastrointestinal formulas differ in that they do not necessarily utilize unique or hydrolyzed protein sources but are designed with highly digestible ingredients to facilitate digestion and absorption of nutrients within a compromised gastrointestinal tract. Veterinary gastrointestinal formulas may also incorporate other dietary modifications such as adjustment of macronutrient distribution, fatty acid composition, and inclusion of dietary fibers and prebiotics.

While nutritional intervention is commonly utilized to manage chronic gastroenteropathy in cats, there have only been a handful of reported studies evaluating the efficacy of nutritional intervention in naturally occurring disease (4–7). Guilford et al. showed that 49% of 55 cats with chronic gastrointestinal disease were responsive to dietary change, and 29% of the 55 demonstrated a food allergy or sensitivity with recrudescence of clinical signs upon diet re-challenge (4). In another study, eight cats with chronic diarrhea and/or vomiting responded to dietary therapy with a hydrolyzed diet after other medical and dietary interventions were unsuccessful (5). Laflamme et al. demonstrated improvement in fecal score in 78.2% of 55 cats with chronic diarrhea fed either a low-fat (23% ME) or high-fat (45.1% ME) dry diet containing common ingredients (6). Additionally, two canned therapeutic gastrointestinal formulas containing common ingredients were evaluated for the management of naturally occurring chronic diarrhea, demonstrating resolution to a normal fecal score in up to 46.7% of 15 cats (7).

While recent studies have focused on chronic diarrhea, less is known regarding dietary intervention with chronic vomiting. Vomiting is the primary clinical sign recognized in feline patients with chronic gastroenteropathy (4, 8, 9). In the Guilford’s study, vomiting was present in 51.4% of the cats, diarrhea in 31.4%, and vomiting and diarrhea in 17.1% (4). Of patient visits to Banfield Pet Hospital^®^ between 2008 and 2012, 1.4% of total feline visits per annum presented with chronic vomiting alone, 0.4% with chronic diarrhea alone, and 0.1% with both chronic vomiting and chronic diarrhea (8). An evaluation of 100 feline cases of chronic small bowel disease suggested that diagnostic evaluation and implementation of dietary management is indicated in cats with vomiting as infrequently as two times per month (9). While the use of elimination diets containing novel and hydrolyzed protein sources has been evaluated (4, 5), to the authors’ knowledge, no studies to date have evaluated the efficacy of feeding a non-specific gastrointestinal therapeutic diet to cats with chronic gastroenteropathy with vomiting included as one of the primary clinical signs.

## Materials and Methods

### Study Population

Cats with chronic vomiting and/or diarrhea were recruited by a multi-center group of primary care veterinarians in the greater Cincinnati region. Cats with at least one episode of vomiting and/or diarrhea per week for a minimum duration of 3 weeks, with no more than three episodes of vomiting daily were included. The vomitus excluded those defined as hairball only but could include hair material if present in addition to food and/or bile. Cats were at least 1 year of age, with a body condition score (BCS) between 2 and 4 on a 5-point scale, and housed exclusively indoors. Cats were excluded if they were fed a veterinary therapeutic diet; had a known food allergy; had a history of undesired weight loss in excess of 10% over the last 2 months; had received antibiotic therapy within the last month; received long-acting immunosuppressive drugs within the past 6 weeks; or had received any immunosuppressive drugs within 3 weeks prior to enrollment.

Cat owners were required to be at least 18 years of age, be the primary caregiver of the cat, be able to monitor the frequency of vomiting and/or diarrhea, and be able to report the daily occurrences *via* a daily automated phone call from an Interactive Voice Response System (IVRS). Cats from multi-cat households (up to four cats maximum) could be included, but only one cat was eligible to participate in the study and all cats in the house-hold were required to be fed the same study diet. Additionally, the owner had to be able to monitor the frequency and occurrence of vomiting and/or diarrhea from the cat participating in the study.

### Clinical Screening

On day 0, all cats underwent a baseline physical examination and blood was submitted for complete blood count, serum chemistry panel, total serum thyroxine concentration (Total T4), feline pancreas-specific lipase (Spec fPL), and serum cobalamin and folate testing. Viral screening consisted of feline coronavirus (FCoV) antibody by indirect fluorescent antibody and feline leukemia virus (FeLV) antigen and feline immunodeficiency virus (FIV) antibody by ELISA. Fecal examination included wet smear and zinc sulfate centrifugation flotation and a feline diarrhea PCR panel to detect *Campylobacter coli, Campylobacter jejuni, Clostridium perfringens* enterotoxin gene, *Cryptosporidium spp*., FCoV, feline panleukopenia virus, *Giardia* spp., *Salmonella* spp., *Toxoplasma gondii*, and *Tritrichomonas fetus*. Cats were excluded if diagnostic tests identified any systemic or infectious disease that could be associated with the clinical signs of vomiting or diarrhea, including, but not limited to FeLV, FIV, hepatic disease, renal disease, and hyperthyroidism. Cats were also excluded if they were panhypoproteinemic, hypoalbuminemic, hypocobalaminemic, or had a Spec fPL ≥5.4 µg/L. Cats that tested positive for FCoV antibody, *C. perfringens* enterotoxin gene, *C. coli*, and FCoV *via* the PCR fecal panel were not excluded from the study. All diagnostic analyses were conducted at a third party commercial laboratory.^1^

### Study Diets

Cats were randomized to be fed one of two commercial veterinary therapeutic dry gastrointestinal formulas, Diet A^2^ or Diet B.^3^ No additional foods or treats were permitted. All investigators and participants were masked to the identity of the diet. Study diets were re-packaged into white bags, sealed, and delivered directly to participating veterinary clinics by plant employees not participating in the study. Nutrient analysis (conducted internally at Iams Analytical Laboratory), caloric distribution, and ingredient composition is summarized in Table 1. Cats were fed according to each product’s feeding guidelines to maintain body weight.

**Table 1:** **Nutrient analysis, caloric distribution, and ingredient composition of study diets fed to 28 cats with chronic gastroenteropathy**.

Nutrient analysis	Diet A	Diet B
Moisture, % AF	7.4	7.8
Protein, % AF	32.0	37.4
Protein, g/1,000 kcal	88.4	98.8
Fat, % AF	13.4	17.9
Fat, g/1,000 kcal	36.9	47.5
Ash, % AF	5.9	6.7
Ash, g/1,000 kcal	16.3	17.6
Crude fiber, % AF	2.2	3.2
Crude fiber, g/1,000 kcal	6.1	8.4
Carbohydrate (NFE), % AF	39.1	27.1
Carbohydrate (NFE), g/1,000 kcal	107.8	71.6
**Caloric distribution**		
Calculated ME (modified Atwater), kcal/kg	3,626	3,782
Protein, % ME	31	35
Fat, % ME	31	40
Carbohydrate, % ME	38	25
**Ingredient composition (product label)**		
**Diet A**	**Diet B**	
Chicken by-product meal, corn meal, corn grits, chicken, dried beet pulp, dried egg product, brewers dried yeast, natural flavor, fructooligosaccharides, potassium chloride, fish oil (preserved with mixed tocopherols, a source of vitamin E), chicken fat (preserved with mixed tocopherols, a source of vitamin E), dl-methionine, calcium carbonate, choline chloride, mannanoligosaccharides, salt, vitamins (vitamin E supplement, niacin, ascorbic acid, vitamin A acetate, calcium pantothenate, biotin, thiamine mononitrate (source of vitamin B1), pyridoxine hydrochloride (source of vitamin B6), vitamin B12 supplement, riboflavin supplement (source of vitamin B2), inositol, vitamin D3 supplement, folic acid), taurine, minerals (zinc oxide, manganese sulfate, copper sulfate, potassium iodide), rosemary extract	Chicken by-product meal, brewers rice, corn gluten meal, whole grain corn, pork fat (preserved with mixed tocopherols and citric acid), powdered cellulose, dried chicken, lactic acid, chicken liver flavor, potassium chloride, choline chloride, dried beet pulp, dl-methionine, calcium sulfate, dried yeast, vitamin E supplement, potassium sulfate, vitamins [l-ascorbyl-2-polyphosphate (source of vitamin C), vitamin E supplement, niacin, thiamine mononitrate, vitamin A supplement, calcium pantothenate, riboflavin, biotin, vitamin B12 supplement, pyridoxine hydrochloride, folic acid, vitamin D3 supplement], iodized salt, minerals (ferrous sulfate, zinc oxide, copper sulfate, manganous oxide, calcium iodate, sodium selenite), taurine, vitamins preserved with mixed tocopherols and citric acid, phosphoric acid, beta-carotene, rosemary extract	

*Nutrient analysis conducted at the Iams Pet Care Analytical Lab; AF, as fed; NFE, nitrogen-free extract, determined as 100% − (% moisture + % protein + % fat + % ash + % crude fiber); ME, metabolizable energy*.

### Study Design

**Figure 1:**
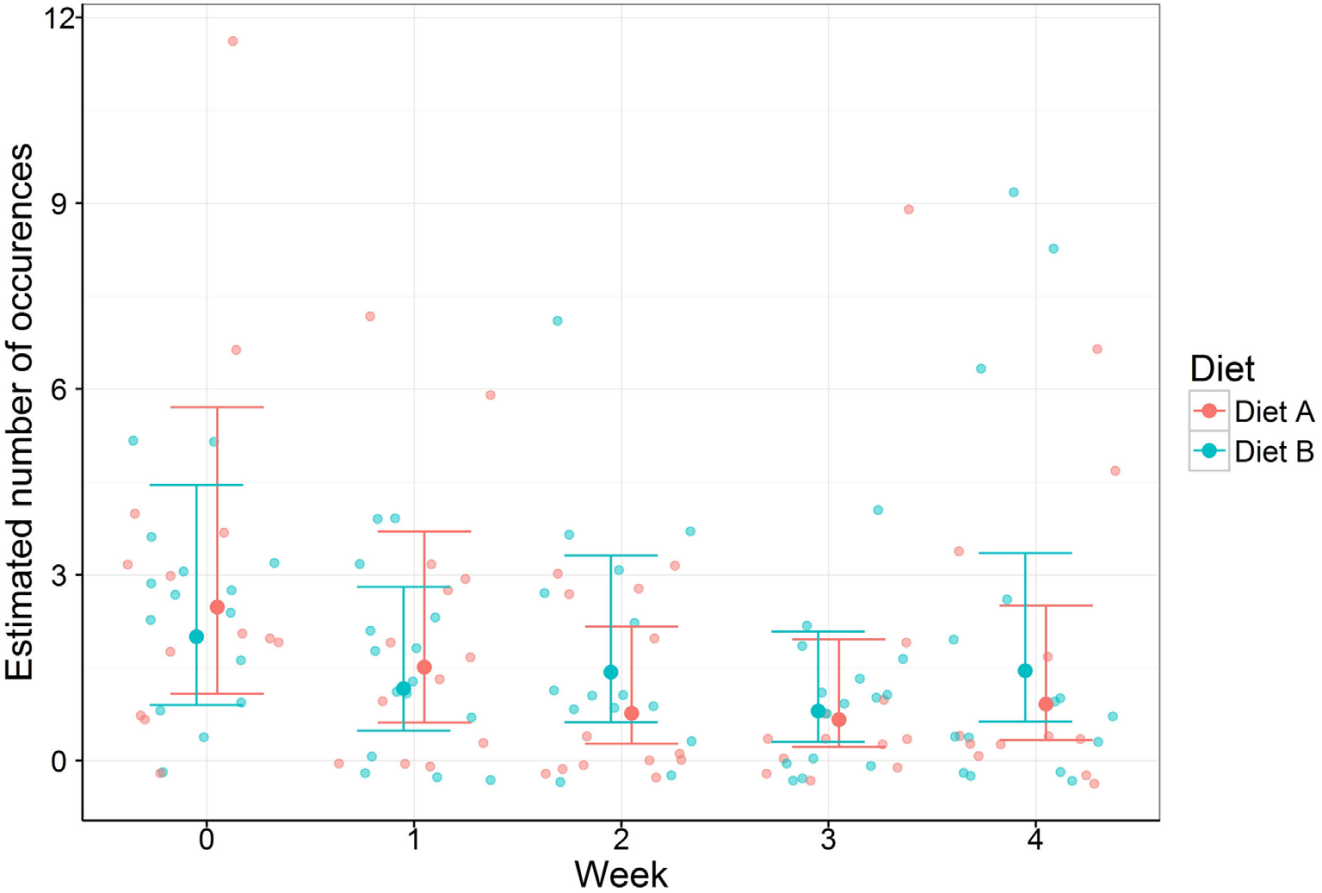
**Vomiting occurrences with 95% confidence intervals in 28 cats with chronic gastroenteropathy fed one of two different veterinary therapeutic diets**.

On day 0, cats that met all of the inclusion criteria were randomized to receive either Diet A or Diet B. The randomization was balanced by site, presence of diarrhea, and the owner reported weekly average number of vomiting and/or diarrhea episodes. Randomization was conducted with an internally developed balance and assignment program through an electronic case record form. Withdrawal criteria included any identified condition that required immediate medical care. Owners were trained to use the feline Fecal Consistency Scale (FCS; Figure 1) and to respond to the daily IVRS phone call. On days 1–28, owners received daily IVRS phone calls to record the number of vomiting episodes and presence of diarrhea over the 24-h period. A diary was provided to track daily observations and to record daily FCS scores. Owners were instructed to call the veterinary clinic if the cat did not consume the food, had a reduced appetite, reduction in body weight, or any other concerns. On day 14, the owner was called by a veterinary technician to confirm that cat was eating well, had not lost weight, and did not have any concerns or significant decline in their clinical condition. On day 28, the cats returned to the clinic for a final examination and diagnostic recheck (CBC, serum chemistry profile, and Spec fPL).

### Statistical Methods

The weekly episodes of vomiting and diarrhea were analyzed with a mixed-effects negative binomial generalized linear model. Fixed effects in the model were week, diet, and the interaction between. The random effects were animal nested within clinic. For vomiting, the change from week 0 to weeks 1–4 for each diet and between diets was tested. For diarrhea, the change from week 1 to weeks 2–4 for each diet and between diets was tested.

The negative binomial models were also used to test for an effect of positive fecal testing for FCoV, *C. perfringens* enterotoxin gene, and FCoV antibody on vomiting and diarrhea occurrences by changing the fixed effects to week and positive/negative and retaining the same contrasts. This same model was altered to have a response of the total vomiting occurrences over the 4 weeks and fixed effects of diet and age. Vomiting was also modeled against the initial Spec fPL values using a negative binomial model, as before, however with the change in vomiting from baseline as the response and the fixed effect of Spec fPL results. The fecal scores were modeled using a linear mixed-effects model with fixed effects of day, diet, and the interaction between and a random effect of cat.

The binary data calculated from the vomiting occurrences was analyzed with a mixed-effects binomial model, using a logit link function. As before, the fixed effects were week, diet, and interaction between. Random effects were animal nested within clinic. The same hypothesis tests were also performed comparing the change between weeks within and between diets.

All contrasts performed using the mixed-effects models were corrected for the multiple comparisons using stepwise correction, to achieve a family wise error rate of 5% within each measure. The hypothesis tests of correlations were corrected using the false discovery rate method of Benjamini–Hochberg, to the 5% level. These analyses were performed in R v3.2.0 (10) statistical software with libraries *lme4* (11) and *multcomp* (12).

## Results

### Study Population

Fifty-six cats were screened to participate in the study. Of the 56 cats, 4 did not meet the initial pre-diagnostic inclusion criteria. On day 0, 52 cats were randomized to 1 of the 2 study diets, of which 17 were found to either violate the inclusion or meet the exclusion criteria based on the results of the diagnostic screening. Seven cats dropped out of the study prior to the day 28 visit. Reasons for dropping out included unwillingness to eat the diet in four cats (two cats fed Diet A and two cats fed Diet B), withdrawn by owner due to continued diarrhea in two cats (one fed Diet A for 1 day and one fed Diet B for 4 days), and one owner declined to participate following screening for one cat. The final study population of 28 cats included 13 cats fed Diet A and 15 cats fed Diet B. One of the cats in the Diet A group was inadvertently given the wrong diet after 2 weeks; therefore, data for this cat were included for only the first 2 weeks of the feeding period.

### Demographic and Baseline Characteristics

Mean age was 8.0 years (SD = 3.9, range 2–15 years) and 6.5 years (SD = 3.6, 1.0–14 years) in cats fed Diets A and B, respectively. Spayed females represented 62% (*n* = 6) and 40% (*n* = 6), while neutered males represented 38% (*n* = 5) and 60% (*n* = 9) of cats fed Diets A and B, respectively. Mean body weight was 4.9 kg (SD = 1.3, range 3.0–7.3) and 4.6 kg (SD = 1.2, range 2.9–7.0); and mean BCS was 3.2 (SD = 0.6, range 2–4) and 3.1 (SD = 0.6, range 2–4) on a 5-point scale in cats fed Diets A and B, respectively. Results of the PCR fecal panel showed that 16 of the 28 cats (Diet A *n* = 9; Diet B *n* = 7) tested positive for *C. perfringens* enterotoxin gene; 1 cat (Diet A *n* = 1; Diet B *n* = 0) tested positive for *C. coli*; and 17 cats (Diet A *n* = 9; Diet B *n* = 8) tested positive for FCoV. Eighteen of the 28 cats (Diet A *n* = 10; Diet B *n* = 8) tested positive for FCoV antibody. There were no statistically differences in the baseline characteristics of the cats between diet groups, and diagnostic screenings showed no clinically meaningful differences.

### Comparison of Dietary Interventions

Cats fed both Diets A and B had a numerical reduction in the mean weekly number of vomiting episodes, with little to no change in weekly diarrhea episodes, over the 4-week period (Table 2). There were no differences between diet groups in the mean weekly number of vomiting or diarrhea episodes (*p* > 0.05). Significant differences were seen in vomiting episodes over time within cats fed Diet A between week 0 and weeks 2–4. These had fold changes in the number of vomiting occurrences of 3.24, 3.74, and 2.72 (indicating reduction of 69.1, 73.3, and 63.2%), respectively, with *p* values of 0.008, 0.003, and 0.029, respectively (Table 3). Changes in vomiting episodes within cats fed Diet B did not reach significance over the 4-week study period (*p* > 0.05) (Table 3). There were no statistical differences in the change of diarrhea episodes over the 4-week study (*p* > 0.05).

**Table 2:** **Estimated means and 95% confidence intervals (CIs) for weekly vomiting and diarrhea episodes in 28 cats with chronic gastroenteropathy**.

Measure	Diet	Week	Estimate	95% CI	
				Lower	Upper
Vomiting	Diet A	0	2.5	1.08	5.71
		1	1.5	0.61	3.70
		2	0.8	0.27	2.16
		3	0.7	0.22	1.96
		4	0.9	0.33	2.51
	Diet B	0	2.0	0.90	4.45
		1	1.2	0.48	2.80
		2	1.4	0.62	3.31
		3	0.8	0.31	2.08
		4	1.5	0.63	3.35
Diarrhea	Diet A	1	0.5	0.17	1.75
		2	0.5	0.16	1.79
		3	0.5	0.14	1.66
		4	0.5	0.14	1.67
	Diet B	1	0.3	0.10	1.20
		2	0.2	0.04	0.80
		3	0.1	0.02	0.69
		4	0.2	0.05	0.90

**Table 3:** **Estimated fold changes, 95% confidence intervals (CIs), and *p* values for significance of change in vomiting over the 4-week study period in 28 cats with chronic gastroenteropathy**.

Diet	Contrast	Fold change	95% CI		*p*-Value
			Lower	Upper	
Diet A	Week 0 vs. 1	1.6	0.73	3.73	0.53
	Week 0 vs. 2	3.2	1.22	8.59	**0.008**
	Week 0 vs. 3	3.7	1.36	10.33	**0.003**
	Week 0 vs. 4	2.7	1.06	6.94	**0.029**
Diet B	Week 0 vs. 1	1.7	0.74	4.01	0.47
	Week 0 vs. 2	1.4	0.63	3.14	0.88
	Week 0 vs. 3	2.5	0.99	6.34	0.06
	Week 0 vs. 4	1.4	0.62	3.06	0.90

*Significant p-values are listed in bold text*.

The vomiting probability also numerically declined in both groups of cats over the 4-week study. At weeks 0–4, cats fed Diet A had a probability of vomiting of 0.98 [95% confidence interval (CI) 0.42–1.00], 0.80 (95% CI 0.19–0.99), 0.3 (95% CI 0.03–0.87), 0.20 (95% CI 0.01–0.82), 0.20 (95% CI 0.01–0.82), respectively. At weeks 0, 1, 2, 3, and 4 cats fed Diet B had a probability of vomiting of 0.96 (95% CI 0.45–1.00), 0.86 (95% CI 0.27–0.99), 0.92 (95% CI 0.35–1.0), 0.78 (95% CI 0.20–0.98), and 0.57 (0.10–0.94), respectively. Statistical differences were seen between time points within cats fed Diet A between week 0 and weeks 2–4, with odds ratios of 0.008, 0.005, and 0.005 respectively (*p* values of 0.038, 0.023, and 0.023) (Table 4). The change in probability of vomiting within cats fed Diet B did not reach significance over the 4-week study period (*p* > 0.05) (Table 4).

**Table 4:** **Estimated fold changes, 95% confidence intervals (CIs), and *p* values for significance of all contrasts for vomiting probability**.

Diet	Contrast	Odds ratio	95% CI		*p*-Value
			Lower	Upper	
Diet A	Week 0 vs. 1	0.08	0.001	5.23	0.46
	Week 0 vs. 2	0.008	0.000	0.84	**0.038**
	Week 0 vs. 3	0.005	0.000	0.61	**0.023**
	Week 0 vs. 4	0.005	0.000	0.62	**0.023**
Diet B	Week 0 vs. 1	0.25	0.009	6.81	0.81
	Week 0 vs. 2	0.47	0.016	13.45	0.99
	Week 0 vs. 3	0.15	0.006	3.97	0.51
	Week 0 vs. 4	0.06	0.002	1.65	0.14

*Significant p-values are listed in bold text*.

Fecal Consistency Scale scores were available for only 4 (31%) cats fed Diet A and 10 (67%) cats fed Diet B. The model of FCS against day and diet over the 4 weeks showed no significant differences between the diets in the change in fecal score (*p* > 0.05). There was no correlation between the frequency of vomiting or diarrhea over the 4-week period and the presence of fecal enteropathogens detected *via* PCR or antibody testing (*p* > 0.05). Similarly, neither Spec fPL results nor age of the cat correlated with the frequency of vomiting over the 4-week period (*p* > 0.05).

## Discussion

While therapeutic veterinary gastrointestinal diets are commonly utilized in veterinary practice for the management of gastroenteritis, this was the first study to evaluate the efficacy of these diets in vomiting cats with chronic gastroenteropathy. The majority of cats in this study presented with chronic vomiting represented in 25 of the 28 cats (89%). There were no statistical differences between dietary treatments in the weekly mean vomiting frequency or change in weekly vomiting frequency over time. However, looking within dietary groups, cats fed Diet A showed a significant reduction in vomiting occurrences in weeks 2–4 compared to baseline, with a 63% reduction in the final week compared to baseline. Cats fed Diet B, while not reaching statistical significance, also showed a numerical reduction from baseline over the study period. Follow-up studies with additional cats are needed to better define the efficacy of Diet B and any potential differences between dietary treatments.

In addition to the change of mean weekly vomiting episodes within treatment groups, it was also of interest to consider the number of cats that reached resolution, or no vomiting episodes, over the weekly period. The probability of vomiting declined during the course of the study, with a significant decline in cats fed Diet A in weeks 2–4, with a 0.2 probability in the final week. Sixty seven percent (*n* = 8) of the cats fed Diet A and 47% (*n* = 7) of the cats fed Diet B had no vomiting episodes at the final week of the study. Of these 15 cats, 3 showed an episodic pattern over the 4-week period, with 1 or more weeks without vomiting followed by a later week with vomiting.

Chronic diarrhea was less common in this study population, with only two cats having concurrent vomiting and diarrhea and three cats with diarrhea alone. It was considered that there may have been a lack of awareness or appreciation of diarrhea in the cats given the absorptive nature of litter that could have made diarrhea less recognizable to owner. The FCS chart included images of stool within litter to help educate and increase awareness of the owners participating in this study; however, overall frequency of diarrhea remained low and did not significantly change over the 4-week period.

Data from Banfield Pet Hospital^®^ visits between 2008 and 2012 showed that older cats are more highly represented among cases of chronic gastrointestinal disease (8). Because of this, it was considered that age may have been associated with more severe clinical disease; however, no significant correlation was observed between age and vomiting occurrence within this study population. Previous studies evaluating diagnostic outcomes of cats with chronic gastroenteropathy have shown that older cats (mean ages ranging from 9.3 to 12.6 years) were more likely to have an underlying neoplastic etiology compared to younger populations (mean age 7.7–8.9 years) (13, 14). The population in this present study was relatively younger (mean age of 7.2 years) and likely had less severe disease than those evaluated in previous studies at tertiary referral practices. Given the risk of underlying neoplastic disease in cats with chronic gastroenteropathy, dietary intervention in conjunction with additional diagnostics is warranted in older patients.

Similar to previous reports, we found a relatively high prevalence of cats that were positive for FCoV antibody and *C. perfringens* enterotoxin gene (15). Cats with a positive status were included within this study population, receiving dietary intervention alone. Similar to a previous report (15), a positive status did not correlate with the occurrence of vomiting or diarrhea over the study period. It should be emphasized that the presence of FCoV antibody and *C. perfringens* toxin gene does not necessarily denote infection nor does it indicate the need to initiate medical therapy. Similarly, antimicrobial therapy for *Campylobacter* spp. is not recommended in animals that are not immunocompromised or showing systemic signs of illness (fever, hemorrhagic diarrhea, abnormal leukogram findings) as it can prolong the carrier state and the diarrhea typically self-resolves with supportive care alone (16).

Both Diet A and Diet B are categorized as therapeutic gastrointestinal diets designed to manage non-specific acute and chronic gastrointestinal disorders. Although both diets share similar indications, there are nutritional differences between the two diets that are worth considering. The macronutrient distribution of protein, fat, and carbohydrate differed between the two formulas. The percent of calories from protein, fat, and carbohydrate were 31, 31, and 38% in Diet A and 35, 40, and 25% in Diet B, respectively (Table 1). While protein level was similar between the two formulas, the fat and carbohydrate contents varied more widely. Reduced dietary fat has been recommended in patients with gastroenteropathy due to potentially impaired fat absorption (7, 8); however, dietary fat was shown to have no difference in clinical response in cats with chronic diarrhea (6). The presence of concurrent chronic pancreatitis has also been an indication for feeding reduced or moderate fat diets in cats with gastroenteropathies (17); however, cats with increased fPL levels were excluded from this study. Carbohydrate malabsorption was previously reported in cats with IBD, but dietary carbohydrates were not shown to negatively impact clinical signs (18). The higher dietary carbohydrate content of Diet A, relative to Diet B, did not appear to negatively impact diet performance and was well tolerated.

In addition to total dietary fat, the fatty acid composition should also be considered. Based on the guaranteed analysis for total omega-3 fatty acids and inclusion of fish oil (Table 1), Diet A was expected to provide greater concentrations of long-chain omega-3 fatty acid. Dietary long-chain omega-3 fatty acids have been shown to modulate inflammation within the body, providing benefit to a variety of inflammatory medical conditions (19). The underlying etiology was not defined in this study; however, IBD is commonly reported in feline gastroenteropathy, and the use of omega-3 fatty acids may have a beneficial effect in modulating the inflammatory response (19). While the duration of this study was relatively short, fatty acid incorporation into intestinal tissues can occur fairly rapidly, with one study showing significantly higher concentrations of eicosapentaenoic acid and docosahexaenoic acid in human colonic tissue following 7 days of supplementation compared to controls (20). Additional studies evaluating diets with titrated levels of long-chain omega-3 fatty acids are required to better understand potential benefits in cats with chronic gastroenteropathy.

Finally, differences in dietary fiber and prebiotic ingredients may have influenced response. Crude fiber analysis was 2.22% in Diet A and 3.16% in Diet B. Total dietary fiber and the soluble and insoluble fractions were not analyzed in this study. These data would have helped to better characterize the dietary fiber composition of the diets; however, ingredient composition provides some insight as to how these two diets differ. Diet A utilized dried beet pulp, fructooligosaccharides, and mannanoligosaccharides, while Diet B utilized powdered cellulose followed by dried beet pulp. Based on the ingredient composition, Diet A would be expected to provide more fermentable fibers and prebiotics that may have promoted an increased production of total intraluminal short-chain fatty acids and positively influenced microbial populations (21–30). While dietary fiber and prebiotics are generally considered to provide benefits primarily within the large intestine and aid in the management of diarrhea, recent studies have demonstrated immune modulating benefits that could have broader benefits, particularly in conditions with an inflammatory nature (31, 32). The time required to see potential clinical benefits is unclear, and clinical improvement related to immunomodulation may require more than 1–2 weeks of dietary intervention. Additional studies are needed to better understand the role of the microbiota in inflammatory gastrointestinal disease and the potential benefits of dietary fibers and prebiotics in animals with chronic gastroenteropathies.

There were several limitations to this study, including the relatively small study population that was below the initial target of 25 cats per treatment group. Additional numbers may have been required to demonstrate a significant difference in Diet B and between the two dietary interventions. This study also relied on a comparison to baseline versus a comparison to a non-therapeutic control formula. A non-therapeutic control would have been ideal for establishing effectiveness of the therapeutic diets; however, due to ethical considerations, all study participants were provided with a therapeutic dietary intervention. Additionally, this study relied on the owner’s recall to establish the baseline weekly vomiting frequency. A recorded baseline would have been preferred; however, it was decided that it was not in the cats’ best interest to delay dietary intervention. Finally, the lack of significance may have been influenced by the variability of clinical disease within individual cats. It was appreciated in this study that while most cats had a progressive decline of vomiting occurrences, some cats had episodic occurrences of vomiting that varied from week to week (Figure 1). The 4-week feeding period was chosen to approximate a reasonable duration for a dietary trial with a gastrointestinal therapeutic formula before additional diagnostic procedures and/or medical treatments would be employed to better characterize and manage the underlying disease. While this study did not follow the cats beyond the 4-week period, it would have been optimal to evaluate this cohort for 4–6 months to identify whether there were recrudescence of clinical signs and any differences between the two diet groups long term.

## Conclusion

Results of this study suggest that gastrointestinal therapeutic diets alone can be effective in the management of cats with chronic vomiting associated with a chronic gastroenteropathy. Cats that do not respond to dietary therapy require further diagnostics and/or medical therapy to adequately characterize and manage the underlying disease. Additionally, cats that do not respond to gastrointestinal formulas may respond to diets formulated with limited novel ingredients or hydrolyzed protein sources.

## Ethics Statement

This study was carried out in accordance with the P&G Pet Care Animal Research Policy, and the protocol was approved by the P&G Pet Care Institutional Animal Care and Use Committee. All cat owners provided written consent to participate in the study.

## Author Contributions

SP contributed to the design, to conducting of the study, to the analysis and interpretation of the results, and to the writing of the manuscript. SM contributed to the design, interpretation of the results, and writing of the manuscript. LD and PK contributed to the design, to the conducting of the study, to the interpretation of the results, and to the review of the manuscript. RH contributed to the statistical analysis, interpretation of the results, and writing of the manuscript.

## Conflict of Interest Statement

SP, LD, and PK were employed by Proctor and Gamble at the time of the study. SP and RH are current associates at Mars, Inc.

## Footnotes

^1^Idexx Reference Laboratory, Memphis.

^2^Diet A—Iams® Veterinary Formula™ Intestinal Plus Low-Residue™ Feline dry.

^3^Diet B—Hill’s® Prescription Diet® i/d® Feline Gastrointestinal Health dry.
